# An EQ-5D-5L Value Set for Vietnam

**DOI:** 10.1007/s11136-020-02469-7

**Published:** 2020-03-27

**Authors:** Vu Quynh Mai, Sun Sun, Hoang Van Minh, Nan Luo, Kim Bao Giang, Lars Lindholm, Klas Goran Sahlen

**Affiliations:** 1 Center for Population Health Sciences, Hanoi University of Public Health, Hanoi, Vietnam; 2 Department of Epidemiology and Global Health, Umeå University, Umeå, Sweden; 3 Research group Health Outcomes and Economic Evaluation, Department of Learning, Informatics, Management and Ethics, Karolinska Institutet, 171 77, Stockholm, Sweden; 4 Hanoi University of Public Health, Hanoi, Vietnam; 5 Saw Swee Hock School of Public Health, National University of Singapore, Singapore, Singapore; 6 Hanoi Medical University, Hanoi, Vietnam

**Keywords:** Value set, Utility, Generic measures, EQ-5D-5L

## Abstract

**Purpose:**

The objective of this study was to develop an EQ-5D-5L value set based on the health preferences of the general adult population of Vietnam.

**Methods:**

The EQ-VT protocol version 2.1 was applied. Multi-stage stratified cluster sampling was employed to recruit a nationally representative sample. Both composite time trade-off (C-TTO) and discrete choice experiment (DCE) methods were used. Several modelling approaches were considered including hybrid; tobit; panel and heteroscedastic models. First, models using C-TTO or DCE data were tested separately. Then possibility of combining the C-TTO and DCE data was examined. Hybrid models were tested if it was sensible to combine both types of data. The best-performing model was selected based on both the consistency of the results produced and the degree to which models used all the available data.

**Results:**

Data from 1200 respondents representing the general Vietnamese adult population were included in the analyses. Only the DCE Logit model and the regular Hybrid model that uses all available data produced consistent results. As the priority was to use all available data if possible, the hybrid model was selected to generate the Vietnamese value set. Mobility had the largest effect on health state values, followed by pain/discomfort, usual activities, anxiety/depression and self-care. The Vietnam values ranged from − 0.5115 to 1.

**Conclusion:**

This is the first value set for EQ-5D-5L based on social preferences obtained from a nationally representative sample in Vietnam. The value set will likely play a key role in economic evaluations and health technology assessments in Vietnam.

**Electronic supplementary material:**

The online version of this article (10.1007/s11136-020-02469-7) contains supplementary material, which is available to authorized users.

## Introduction

Thanks in part to advances in medicine and public health, Vietnamese people live longer, though clearly not all years are spent in full health [1]. In such situations, a summary measure, such as quality-adjusted life years (QALYs), which combines both the quality (health status) and quantity (life years) of health, can be a useful tool for decision-makers involved in health technology assessment (HTA) [2, 3]. HTA guidelines are currently being developed for Vietnam and, since 2018, the Ministry of Health has required HTA to be performed for any new drugs intended for inclusion in health insurance packages [4]. QALYs will be considered an important HTA outcome in Vietnam, in line with HTA guidelines in other countries [5].

To operationalize the QALY concept, a means of assigning quality weights to the health states of interest is required [6]. Two important issues need to be addressed when deriving such preference weights. The first is the perspective of the valuation, i.e. whose value to use. Values can be obtained from patient groups (patient values) or from representative samples of the general population (social values) [7–10]. The second important issue is which method to use. Methods commonly used to derive preference weights for health states include time trade-off, standard gamble and rating scales [6]. Recently, discrete choice experiments have become an increasingly popular means of generating such preference weights [11]. The use of different valuation methods and perspectives will lead to different values for health states.

Nevertheless, measuring preferences is a time-consuming and complex task. A widely used alternative is to bypass the measurement task using pre-scored multi-attribute health status classification systems [6]. The three most commonly used systems are the Health Utility Index (HUI), EQ-5D from the EuroQol Group and the Short Form 6D (SF-6D) [6]. The EQ-5D instrument is a recommended method for deriving health state preference weights in many countries, including Australia [12], the UK [13] and several other European countries [14]. In Vietnam, the EQ-5D and SF-6D are mostly applied relative to HUI, and it is likely that the EQ-5D instrument will be recommended as the preferred preference-weighted measure in the Vietnamese national HTA guidelines.

The EQ-5D instrument comprises a descriptive system and a visual analogue scale (EQ-VAS). The descriptive system classifies health on five dimensions: mobility, self-care, usual activities, pain/discomfort and anxiety/depression. Within each dimension, respondents are asked to describe their current health using either three (no problems, some/moderate problems, extreme problems/unable to/confined to bed) or five (no problems, slight problems, moderate problems, severe problems and unable to/extreme problems) levels of severity. This gives rise to two different versions of EQ-5D labelled, respectively, the EQ-5D-3L and the EQ-5D-5L. The EQ-VAS is common to both versions of EQ-5D and is a hash-marked scale ranging from 0 to 100 where 0 represents the worst imaginable health and 100 the best imaginable health. EQ-5D value sets are sets of preference weights (or utilities) which can be applied to all health states generated by a given version of EQ-5D (EQ-5D-3L or EQ-5D-5L).

Though the EQ-5D-3L has been applied in economic evaluations of healthcare services in Vietnam, i.e. people with disability [15] and adolescent reproductive healthcare education [16], since the EQ-5D-5L was introduced in 2012 it has been used more frequently, for example, in studies of people living with HIV [17], the elderly [18], people with non-communicable diseases [19, 20] and young people suffering from internet addiction [21].

Despite the increasing use of EQ-5D in Vietnam [22], no population norm has been established, a country-specific value set is still lacking and studies carried out to date have had to use value sets from Korea [23], Thailand [24] or China [25]. Although values sets from other countries can be used in situations in which no national value set is available, the availability and use of country-specific EQ-5D value sets should be considered best practice in the future [26]. In light of the development of national HTA guidelines for Vietnam, there is a need for a country-specific EQ-5D value set. The aim of this study is to derive a value set based on societal preferences for EQ-5D-5L health states in Vietnam.

## Methods

This study followed a standardized protocol developed by the EuroQol Group (EQ-VT 2.1 in Vietnamese). Fieldwork was conducted between 20 November and 25 December 2017. Trained interviewers carried out face-to-face interviews. Data upload and quality control (QC) were performed daily.

### Study population

Study participants were Vietnamese, over 18 years of age, who were able to read and understand the study questions. Participants were informed about the study and provided their written consent to participate. The study was conducted in six provinces, representing six different geographical regions (Northern mountains, the Red River delta, the Highlands, Central Coast, the South-East and the Mekong river delta). The sample size of the original study was 1200 participants, as per standardized protocol recommendations for the minimum sample size for a valuation study [27, 28]. A multi-stage stratified cluster sampling method was applied. Six provinces, one in each region, were purposefully selected to reflect the average socio-economic level in the area. In the next stage, one urban and one rural cluster were chosen randomly in each province. In the final stage, respondents were recruited from relevant clusters using a probabilistic quota-based method. The quota was set for age groups (18–29 years, 30–44 years, 45–59 years and 60 + years) and sex, based on the Vietnamese general population structure in 2017 [29]. For details of the study sampling frame, please refer to Table 1 in the online supplementary materials. Recruitment was at the level of households, using a door-to-door approach.

**Table 1:** Study sample’s and Vietnam general population’s characteristics

Variables	Study Sample (n = 1200) n (%)	Vietnamese population* (N = 92,695 Mio.) N (%)
Socio-economic Regions		
Central Highland	80 (6.67)	5691 (6.14)
Mekong River Delta	230 (19.17)	17,705 (19.10)
Northern Midland and Mountainous	146 (12.17)	11,958 (12.89)
North Central and Central Coastal	259 (21.58)	19,837 (21.39)
Red River Delta	270 (22.5)	21,134 (22.79)
South-East	215 (17.92)	16,407 (17.69)
Residence		
Urban	425 (35.42)	31,980 (34.50)
Rural	775 (64.58)	60,715 (65.50)
Age group		
18–29	410 (34.17)	31,019 (33.46)
30–44	389 (32.42)	30,112 (32.49)
45–59	257 (21.42)	19,976 (21.55)
60 +	144 (12.00)	11,588 (12.50)
Gender		
Male	588 (49.00)	45,699 (49.30)
Female	612 (51.00)	46,996 (50.70)
Marital status		
Currently married	873 (72.75)	63,218 (68.20)
Others	326 (27.17)	29,477 (31.80)
Missing	1 (0.08)	
Poverty		
Poor and near poor**	77 (6.42)	6489 (7.00)
Non-poor	1123 (93.58)	86,206 (93.00)
Education status		
Lower than primary school	41 (3.42)	NA
Primary school	167 (13.92)	NA
Completed secondary school	370 (30.83)	NA
Completed high school	313 (26.08)	NA
University and higher	307 (25.58)	NA
Missing	2 (0.17)	
EQ-5D-5L self-reported health		
Perfect health	652 (54.33)	NA
Problems at any level on Mobility	116 (9.67)	NA
Problems at any level on Self-care	21 (1.75)	NA
Problems at any level on Usual activities	57 (4.75)	NA
Problems at any level on Pain/discomfort	412 (34.33)	NA
Problems at any level on Anxiety/depression	235 (19.58)	NA
Mean VAS (SD)	81.08 (13.37)	NA

*Data from Vietnam General Statistic Book 2016; **Poverty level was based on Vietnam official poverty line

### Valuation technique

Two valuation techniques were used to obtain health state preferences: (1) composite time-trade-off (C-TTO), with an experimental design incorporating ten blocks of ten health states each, and (2) discrete choice experiments (DCE), in which the experimental design comprised 28 blocks of seven pairs each. Detailed descriptions of the valuation protocol can be found elsewhere [27]. The C-TTO is different from the traditional time trade-off method as the traders are given a lead time of ten more years to trade if they decide that they would prefer to be dead at the start of the trade-off process (the case of worse than dead). Details of the two elicitation methods have been published elsewhere [30–32].

### Quality control

Quality Control tool version 2.5, provided by the EuroQol Group, was employed to mitigate the effect of interviewer bias [31]. The QC tool flags interviews in which anomalies are detected, for example, interviews that are conducted unrealistically fast, which do not introduce the “worse than dead” case, or which show clear logical inconsistency. Interviewers with flagged interviews were re-trained and also invited to observe and reflect on how their colleagues conducted the interviews. Daily discussions between supervisors and interviewers were conducted to bolster the quality control process. In parallel, the research team communicated twice weekly with the EuroQol Group’s scientific group to discuss the QC reports.

### Interviewer training

A two-stage interviewer training procedure was followed. In the first stage, training for research team members was provided by the EuroQol Group following an existing training protocol [27]. In the second stage, the trained research team members provided training to twelve candidate interviewers based on the same protocol. The twelve candidates were recruited from students who had recently graduated from the Hanoi University of Public Health. The candidates practiced interviewing each other during a class-based training session and then performed real interviews during the pilot study in the Duc Thang ward, an urban residential area near the university. The quality of the pilot interviews was evaluated using the QC tool. A meeting was held between candidates and supervisors to obtain feedback and discuss difficulties encountered during the interviews. After the pilot study, ten interviewers were selected to participate in the fieldwork.

### Data collection

The data collection form comprised four main sections. Respondents first provided background demographic information before completing the EQ-5D-5L to provide information on their current health status. At this point, participants were guided through five practice examples of the C-TTO task before being asked to value their ten randomly ordered EQ-5D-5L health states. Finally, they completed seven DCE tasks. After completing the ten C-TTO valuations, participants were shown the rank ordering of those states based on their responses to the task and any states they considered to be disordered were flagged (feedback module).

**Fig. 1:**
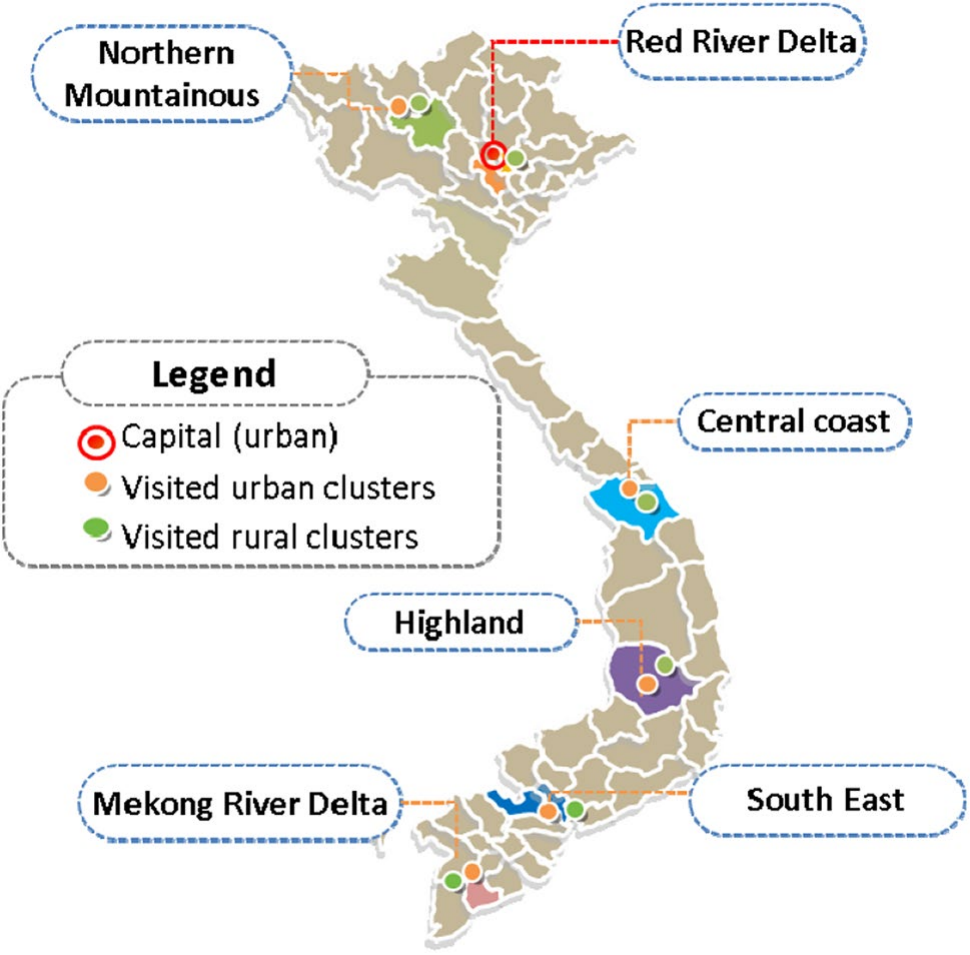
Map indicating regions sample was drawn from for the EQ-5D-5L valuation study in Vietnam

We made some adjustments to the standard EQ-VT protocol to take account of specific circumstances for this type of survey in Vietnam. Firstly, addressing questions directly to someone in relation to illness or being dead in Vietnam can be considered insensitive and, in fact, during the pilot study, the sensitiveness of the topic for both interviewers and respondents became apparent. Interviewers were therefore directed to ask respondents how they thought someone like them (e.g. same age, sex, socio-economic status, etc.) would trade-off time in the C-TTO tasks, instead of the respondents being asked how they would trade-off time themselves. Secondly, our observations from the pilot study suggested that elderly people often felt tired after spending a long time working at a screen in the C-TTO tasks (30 min or more) and they did not completely focus on the next tasks. Instead of carefully comparing the two given health states to complete the DCE tasks, elderly respondents were likely to provide random responses. To improving their concentration, a visual aid in the form of a coloured card was given along with the original visualization of the DCE task on the computer screen. The visual aid included five separate pieces of rectangular paper, printed in five different shades of yellow from lighter to darker according to five levels of severity. Interviewers would use these cards to compare the difference in the colours of options A and B of the pair. For details of the coloured card, Fig. 1 in the online supplementary materials can be consulted.

### Data analysis

Both descriptive statistics and modelling were conducted using Stata software version 15 from the Stata Corporation [33]. Means, standard deviations and 95% confidence intervals were used for continuous variables; frequencies and percentages were used to describe categorical variables.

Data modelling was developed by employing the utility decrement (disutility) as the dependent variable for the C-TTO data and a binary variable 0/1 representing whether state A was chosen vs. state B for the DCE data. We used two sets of independent variables, known as regular dummies and incremental dummies. Both sets comprised four levels to describe health (from “slight problems” to “extreme/unable to do”) for each of the five health dimensions (mobility, self-care, usual activities, pain/discomfort and anxiety/depression). The difference between both is that while regular dummies represent movements from no problems to any other specific level, the incremental dummies represent movements between consecutive levels.

The DCE design includes 10 pairs that are manually added to the experimental design. Oppe and van Hout described this as follows: “We wanted to make sure that 10 very mild pairs would be included in the DC design. Therefore, we fixed these 10, and generated the remaining 186 ones using a design algorithm” [28]. The problem occurs when the observed choice probabilities for these 10 pairs are extreme (> 85%). It tells the model that the distance between the two health states is infinite, causing bias in the model estimations. For this reason, we checked whether the probabilities of these 10 pairs were extreme and we excluded these 10 pairs from our analysis if they were extreme.

### Model construction

Several models were tested to take into account different characteristics of the existing data, i.e. (1) the use of two different valuation methods and the desire to maximize the use of the available data led to the testing of hybrid models; (2) because the composite TTO task does not allow for values lower than − 1 while, theoretically, they could be lower, Tobit models were tested to account for the censored nature of C-TTO data; (3) panel Tobit model (random intercepts model) was tested because of the multiple responses from the same respondent; (4) heteroscedastic models were tested because variance can differ across health states. To compare the C-TTO and DCE model results, the coefficients of the DCE model were rescaled using the rescaling parameter of the TTO model estimations [34]. Further details of the modelling approach are available elsewhere [35, 36].

### Model selection

We first estimated separately an original Tobit, a heteroscedastic Tobit and a panel Tobit model using the C-TTO data and a Logit model using the DCE data. Then we checked whether it was sensible to combine the C-TTO and DCE data using scatter plots to plot predictions of C-TTO models versus predictions of the DCE Logit model. The correlation between the rescaled DCE Logit model and the C-TTO models was tested prior to the hybrid model construction. Next, we estimated hybrid models in case that the presence of C-TTO and DCE data was feasible in a single estimation. The selection of the best-performing model was based on both the consistency of the results produced (i.e. the model which minimized inconsistent orderings or results in the final algorithm) and the degree to which models used all the available data.

## Results

### Data cleaning

Of the 1299 individuals invited to participate, 64 declined (4.9%) and 35 produced incomplete interviews (2.7%). After excluding refusals, incomplete and pilot interviews, data from 1200 respondents were included for analysis. A total of 363 participants in our study had inconsistent responses. However, after removing the flagged health states in the feedback module, this number was reduced to 245. This means that the feedback module was helpful in our study for improving data quality. After checking the observed choice probabilities, we found that the ten manually added DCE pairs had extreme choice probabilities in some of them (see Table II in the supplementary materials). Thus, we excluded the ten pairs from the analysis.

### Sample characteristics

Table 1 shows the study sample’s characteristics in comparison with the general population of Vietnam. Overall, the study sample matched the structure of the Vietnamese general population on the variables being considered. Almost two-thirds of the sample lived in rural areas (64.58%), which is similar to the national statistics. The proportion of males and females was equally distributed and most of the participants were of working age (18–49 years, 88%), which also matched the national population structure. In EQ-5D-5L, 54.33% of respondents reported no problems on any dimension (i.e. were in health state 11111). Respondents most often reported problems in the pain/discomfort dimension (34.33% of the sample), followed by anxiety/depression (19.58%), mobility (9.67%), usual activities (4.75%) and self-care (1.75%). Of all respondents who had problems in any dimension (548 people), 93.07% of them were reported having “slight” problems for at least one dimension. Only two individuals (0.36% of respondents who had problems in any dimension) reported “extreme” problems on any dimension. The mean VAS score was 81.08.

### Model selection

Table 2 presents the incremental disutility predictions from tested C-TTO models including the Tobit, heteroscedastic Tobit (hetTobit), Panel Tobit and the rescaled DCE Logit models. None of the tested C-TTO models generated consistent results. The disordered parameters were reported at a moderate level on self-care for hetTobit. The Tobit and Panel Tobit produced two inconsistent parameters at a moderate level of mobility and self-care. In contrast, weights estimated using the DCE Logit model were consistent.

**Table 2:** Incremental disutility predictions from the C-TTO and DCE models

	Tobit (C-TTO)		hetTobit (C-TTO)		Panel Tobit (C-TTO)		Logit (DCE)	
	Coeff	*P*-value	Coeff	*P*-value	Coeff	P-value	Coeff	*P*-value
Mobility (MO)								
Disutility MO1–MO2	0.043	0.001	0.052	0.000	0.035	0.000	0.087	0.000
Disutility MO1–MO3	**− 0.001**	0.964	0.004	0.779	**− 0.006**	0.611	0.013	0.218
Disutility MO1–MO4	0.128	0.000	0.130	0.000	0.141	0.000	0.131	0.000
Disutility MO1–MO5	0.153	0.000	0.132	0.000	0.155	0.000	0.168	0.000
Self-care (SC)								
Disutility SC1–SC2	0.071	0.000	0.080	0.000	0.062	0.000	0.021	0.069
Disutility SC1–SC3	**− 0.001**	0.958	**− 0.010**	0.403	**− 0.003**	0.817	0.009	0.423
Disutility SC1–SC4	0.093	0.000	0.092	0.000	0.109	0.000	0.106	0.000
Disutility SC1–SC5	0.096	0.000	0.091	0.000	0.088	0.000	0.083	0.000
Usual activity (UA)								
Disutility UA1–UA2	0.064	0.000	0.076	0.000	0.059	0.000	0.045	0.000
Disutility UA1–UA3	0.017	0.212	0.008	0.484	0.019	0.089	0.006	0.581
Disutility UA1–UA4	0.103	0.000	0.118	0.000	0.095	0.000	0.126	0.000
Disutility UA1–UA5	0.109	0.000	0.079	0.000	0.121	0.000	0.125	0.000
Pain/Discomfort (PD)								
Disutility PD1–PD2	0.066	0.000	0.076	0.000	0.058	0.000	0.101	0.000
Disutility PD1–PD3	0.066	0.000	0.049	0.001	0.068	0.000	0.059	0.000
Disutility PD1–PD4	0.149	0.000	0.143	0.000	0.154	0.000	0.110	0.000
Disutility PD1–PD5	0.117	0.000	0.146	0.000	0.114	0.000	0.093	0.000
Anxiety/depression (AD)								
Disutility AD1–AD2	0.069	0.000	0.068	0.000	0.063	0.000	0.060	0.000
Disutility AD1–AD3	0.034	0.023	0.028	0.028	0.029	0.016	0.055	0.000
Disutility AD1–AD4	0.055	0.000	0.064	0.000	0.062	0.000	0.062	0.000
Disutility AD1–AD5	0.089	0.000	0.080	0.000	0.087	0.000	0.053	0.000
Utility for the worst health state (55,555)	− 0.520		− 0.509		−0.511		− 0.514	

The coefficients shown in the table reported incremental dummies of each model. *MO* Mobility, *SC* Self-care, *UA* Usual activities, *PD* Pain/discomfort, *AD* Anxiety/depression. MO1-AD1 = No problem; MO2-AD2 = Slight problem; MO3-AD3 = Moderated problems; MO4-AD4 = Severe problems; MO5-AD5 = Extreme problems. Bolded coefficients reported logical inconsistent. Coefficients in DCE model were rescaled using C-TTO information to be anchor in the 0–1 scale

**Fig. 2:**
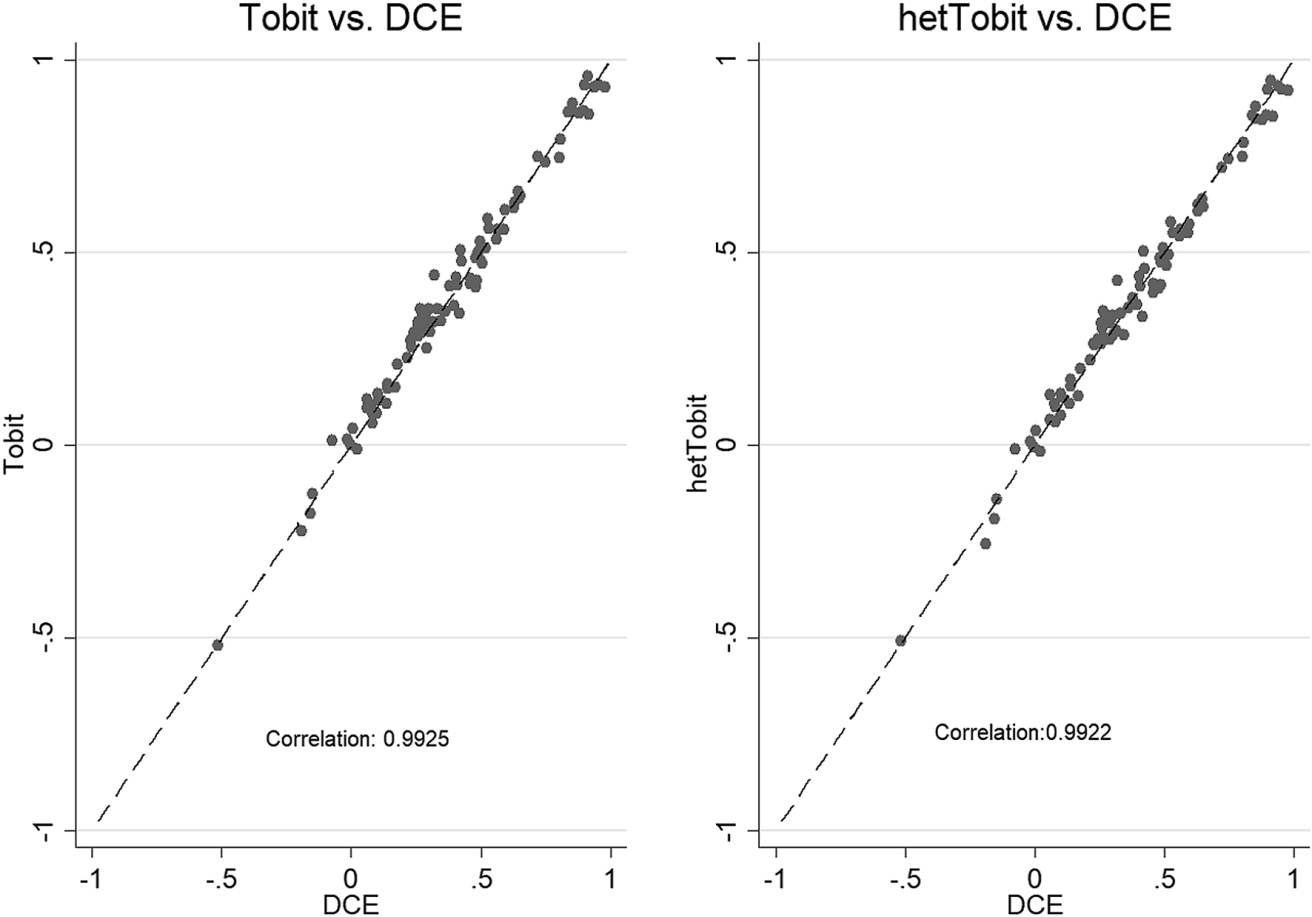
Scatter plots of C-TTO vs DCE model predictions

Figure 2 presents strong agreements between the weights predicted by the DCE Logit model versus the C-TTO regular Tobit and C-TTO hetTobit model, respectively. The high correlations thereby support the single estimation [37]. Then, we constructed the regular censored hybrid model (Hybrid model) and the censored hybrid heteroscedastic model.

The censored hybrid heteroscedastic model led to disordered parameters in some cases, whereas those parameters produced by the Hybrid model were consistent. Thus, we had to choose between the rescaled DCE Logit model or the regular censored hybrid model that uses all available data. As one of our priorities was to use all available data if possible, we selected the hybrid model as the best candidate for generating the Vietnamese value set.

**Fig. 3:**
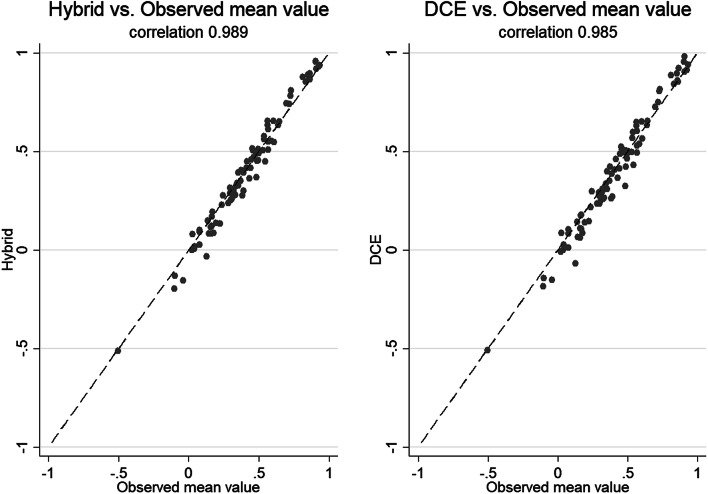
Scatter plots of observed mean value vs DCE and Hybrid model predictions

Figure 3 illustrates the matching between the observed mean values (recorded from C-TTO tasks) and the corresponding DCE Logit model and Hybrid model for the set of health states included in the TTO design. Both values generated from the Hybrid and DCE model were strongly correlated with the observed mean values. The values from the Hybrid model, however, appeared to be slightly closer to the observed mean values than those from the DCE model. For details regarding the distribution and descriptive statistics of the observed mean C-TTO values, please refer to Fig. 2 and Table 3, respectively, in the online supplementary materials.

**Table 3:** Disutility predictions from the selected model (regular censored Hybrid model)

Incremental dummies				Regular dummies (Final model)		
	Coeff	P-values	SE		Coeff	SE
Mobility (MO)				Mobility (MO)		
Disutility MO1–MO2	0.0692	0.000	.0072	Disutility MO1–MO2	0.0692	0.007
Disutility MO2–MO3	0.0093	0.281	.0087	Disutility MO1–MO3	0.0785	0.008
Disutility MO3–MO4	0.1279	0.000	.0090	Disutility MO1–MO4	0.2064	0.008
Disutility MO4–MO5	0.1697	0.000	.0089	Disutility MO1–MO5	0.3761	0.008
Self-care (SC)				Self-care (SC)		
Disutility SC1–SC2	0.0428	0.000	.0073	Disutility SC1–SC2	0.0428	0.007
Disutility SC2–SC3	0.0032	0.710	.0086	Disutility SC1–SC3	0.0460	0.008
Disutility SC3–SC4	0.1012	0.000	.0091	Disutility SC1–SC4	0.1470	0.008
Disutility SC4–SC5	0.0841	0.000	.0085	Disutility SC1–SC5	0.2311	0.008
Usual activity (UA)				Usual activity (UA)		
Disutility UA1–UA2	0.0464	0.000	.0072	Disutility UA1–UA2	0.0464	0.007
Disutility UA2–UA3	0.0123	0.130	.0081	Disutility UA1–UA3	0.0587	0.008
Disutility UA3–UA4	0.1148	0.000	.0086	Disutility UA1–UA4	0.1735	0.008
Disutility UA4–UA5	0.1254	0.000	.0089	Disutility UA1–UA5	0.2989	0.008
Pain/Discomfort (PD)				Pain/Discomfort (PD)		
Disutility PD1–PD2	0.0839	0.000	.0068	Disutility PD1–PD2	0.0839	0.007
Disutility PD2–PD3	0.0682	0.000	.0084	Disutility PD1–PD3	0.1521	0.008
Disutility PD3–PD4	0.1179	0.000	.0088	Disutility PD1–PD4	0.2700	0.008
Disutility PD4–PD5	0.0965	0.000	.0095	Disutility PD1–PD5	0.3666	0.009
Anxiety/Depression (AD)				Anxiety/Depression (AD)		
Disutility AD1–AD2	0.0638	0.000	.0068	Disutility AD1–AD2	0.0638	0.007
Disutility AD2–AD3	0.0489	0.000	.0085	Disutility AD1–AD3	0.1126	0.008
Disutility AD3–AD4	0.0588	0.000	.0087	Disutility AD1–AD4	0.1713	0.008
Disutility AD4–AD5	0.0675	0.000	.0086	Disutility AD1–AD5	0.2388	0.008
Utility value at health state:						
11111 (full health)		1				
12111 (second best health state)		0.9573				
11211		0.9536				
11112		0.9362				
21111		0.9308				
11121		0.9161				
55555 (the worst health state)		− 0.5115				

For instance, value of the health state 12345 is calculated as: 1 − (MO1) − (disutility SC1 − SC2) − (disutility UA1 − UA3) − (disutility PD1 − PD4) − (disutility AD1 − AD5) = 1 − (0) − (0.0428) − (0.0587) − (0.2700) − (0.2388) = 0.3897

### Final model

Table 3 shows the disutility coefficients from the Hybrid model (final model). In terms of the predicted values for 3125 health states, the values ranged from 1 to − 0.5115. The percentage of negative values in the selected value set was 8.3%. The largest disutility weights were observed for the mobility dimension, ranging from 0.0692 for “slight problems” to 0.3761 for “unable to walk”. However, the disutility weights associated with pain/discomfort were of similar importance (0.3666 for extreme problems). The smallest disutility weights were in self-care (0.0428 for “slight problems” to 0.2311 for “unable to”), though disutility weights in the anxiety/depression dimension were similar (0.2388 for “extreme problems”). Disutility weights from this Hybrid model were used to calculate values for all health states in the Vietnamese EQ-5D-5L value set. For example, the value of the health state 12345 is calculated as: 1—(MO1)—(disutility SC1–SC2)—(disutility UA1–UA3)—(disutility PD1–PD4)—(disutility AD1–AD5) = 1– (0) − (0.0428) − (0.0587) − (0.2700) − (0.2388) = 0.3897. The value for the second best health state (12,111) was 0.9573 and the value for the worst health state (55,555) was − 0.5115.

## Discussion

This study has provided a value set based on societal preferences for EQ-5D-5L health states in Vietnam. Values were obtained from a nationally representative sample using the latest version of EQ-VT. The value set can be used for QALY calculations based on the EQ-5D-5L descriptive system and will be a useful tool for local policymakers and HTA researchers.

As previously noted, to date, no national EQ-5D value set was available for use in Vietnam. Previous studies using EQ-5D in Vietnam had adopted value sets from Thailand [24], Korea [23] or China [25]. However, such approaches risk not reflecting actual health preference of the Vietnamese, as well as failing to have a standard EQ-5D value set in Vietnam. In fact, the approach to modelling can vary when developing national value sets. While Vietnam and Thailand used hybrid models to generate their final value sets, in Korea and China, only TTO data were used in the final models. Likewise, differences have been observed in the values assigned to the worst health state (55555), ranging from − 0.5115 in Vietnam to − 0.3910 in Thailand, − 0.4212 in China and − 0.066 in Korea [23–25].

Overall, the Vietnamese place the most weight on mobility and pain/discomfort dimension, which is in line with other published EQ-5D-5L value sets in Asia [38]. When dimensions are ranked according to the disutility corresponding to the level “unable to/extreme”, the Usual activities was ranked third in Vietnam, which means it is given higher weight than in many other countries [38]. A possible explanation is that 57% of Vietnamese employees are informal workers and have limited access to social welfare [29]. Thus, experiencing problems performing usual activities may have a considerable impact on their ability to make a living. Likewise, in contrast to Western countries such as Ireland [39], the Netherlands [40], Germany [41] and the UK [42], in which anxiety/depression was assigned the largest or second largest weight, it was only ranked fourth in Vietnam. This is in line with studies from a number of other Asian countries/regions such as Hong Kong [43], Indonesia [44] and South Korea [23]. The difference could be due to the fact that people in western countries are more aware of mental health [45] and more likely to acknowledge anxiety/depression as a health problem [46].

Differences such as these show why it is preferable for Vietnam to have its own value set. Furthermore, the availability of a local, standardized national value set increases the credibility of results obtained using EQ-5D-5L and of the outcome of cost-effectiveness analysis using country-specific data.

Due to the sensitivity of discussing “dead” in Vietnamese culture, the “third person” approach was employed in the C-TTO exercise. This created a comfortable environment and helped establish a good relationship between interviewers and respondents, as well as reduce the risk of respondents abandoning the interview. On the other hand, it is not clear how the use of the “third person” approach might affect values and further research is necessary to explore this [47].

We decided that the most optimal method of estimating a value set in Vietnam was via the hybrid model, which has been adopted in many other countries [38]. An argument for using the hybrid model is that combining the results from the C-TTO and DCE exercises maximizes the use of all available data. It has also been suggested that both TTO and DCE tap into the same preference structure. Thus, adding DCE responses could improve the ability to predict TTO responses [48]. However, the fact that they are very different valuation methods has led others to argue that there is no robust theoretical justification for combining them in the same model [6]. Despite the controversy of combining TTO and DCE data, Ramos-Goni and colleagues have supported the idea of integrating the two types of data (hybrid approach) in developing models for the EQ-5D-5L valuation studies in the case this approach produces more precise estimates [37]. In the present study, we preferred the regular censored hybrid model because it provided consistent estimates and used both types of available data, which were our priorities when choosing between models.

There are some notes in the study. The first note is our modification to the standard protocol for the EQ-5D-5L valuation study. That may affect to any purpose of cross-country comparison involving the Vietnam value set. Additionally, the use of DCE cards has not been systematically recorded, which could potentially bias this study. Another potential limitation of our study is the possibility of interviewer bias. Our efforts to reduce interviewer bias included re-training and daily group discussions to help interviewers improve their interviewing skills. Also, the fact that the C-TTO is a complicated exercise can lead interviewers to focus on younger respondents because they find the task somewhat easier. The interviewer biases was avoided by using the QC tool and online electronic reporting, which provided real-time updates on participants by age, sex, and place of residence.

## Conclusion

This study presents the first value set for EQ-5D-5L based on social preferences obtained from a nationally representative sample in Vietnam. The results of this study will likely play a key role in economic evaluations and health technology assessments in Vietnam in the future and will be of great value to local policymakers.

## Electronic supplementary material

Below is the link to the electronic supplementary material.

Electronic supplementary material 1 (DOCX 18 kb)

Electronic supplementary material 2 (DOCX 25 kb)
