# Supplementary material for: An EQ-5D-5L Value Set for Vietnam
Source: Qual Life Res. 2020 Mar 27;29(7):1923–33. doi: 10.1007/s11136-020-02469-7 (PMC7295839; doi:10.1007/s11136-020-02469-7)
Supplement: Supplementary file 1 — Electronic supplementary material 1 (DOCX 18 kb) [file 11136_2020_2469_MOESM1_ESM.docx]

## Figure I. An example of the paper-based-color-card used in DCE tasks.

*Notes: The card was not showing in computer, the card was paper-based. Five shades of yellow had been used to present five severity levels. Each shade was cut separately for the interviewer’s convenience, they would pick any shade according to the DCE pairs they were working on.*

## Figure II: The distribution of mean observed C-TTO values for 86 health states

**
