# Supplementary material for: An EQ-5D-5L Value Set for Vietnam
Source: Qual Life Res. 2020 Mar 27;29(7):1923–33. doi: 10.1007/s11136-020-02469-7 (PMC7295839; doi:10.1007/s11136-020-02469-7)
Supplement: Supplementary file 2 — Electronic supplementary material 2 (DOCX 25 kb) [file 11136_2020_2469_MOESM2_ESM.docx]

## Table I. The national population fraction and sample frame

|  | **Urban - Male** | | | | | | | | | | | | |
| --- | --- | --- | --- | --- | --- | --- | --- | --- | --- | --- | --- | --- | --- |
|  | %Pop of region | Sample size | %urban in region | Sample in urban cluster | %male | Sample male | %age 18-29 | Sample male age 18-29 | %age 30-44 | Sample male age 30-44 | %age 45-59 | Sample male age 45-59 | Sample male age 60+ |
| Hanoi | 23% | 272 | 34% | 92 | 49% | 45 | 40% | 18 | 29% | 13 | 20% | 9 | 5 |
| Hoa Binh | 13% | 154 | 18% | 28 | 50% | 14 |  | 6 |  | 4 |  | 3 | 1 |
| Phu Yen | 21% | 257 | 28% | 73 | 49% | 36 |  | 14 |  | 10 |  | 7 | 5 |
| Dak Lak | 6% | 74 | 30% | 22 | 51% | 11 |  | 4 |  | 3 |  | 2 | 2 |
| Ho Chi Minh | 18% | 212 | 63% | 134 | 49% | 65 |  | 26 |  | 19 |  | 13 | 7 |
| Dong Thap | 19% | 231 | 25% | 58 | 50% | 29 |  | 12 |  | 8 |  | 6 | 3 |
| **Total sample** |  | **1200** |  | **407** |  | **200** |  | **80** |  | **57** |  | **40** | **23** |
|  | **Urban - Female** | | | | | | | | | | | | |
|  | %Population of region | Sample size | %urban in region | Sample in urban cluster | %female | Sample female | %age 18-29 | Sample female age 18-29 | %age 30-44 | Sample female age 30-44 | %age 45-59 | Sample female age 45-59 | Sample female age 60+ |
| Hanoi | 23% | 272 | 34% | 92 | 51% | 47 | 35% | 16 | 31% | 15 | 22% | 10 | 6 |
| Hoa Binh | 13% | 154 | 18% | 28 | 50% | 14 |  | 5 |  | 4 |  | 3 | 2 |
| Phu Yen | 21% | 257 | 28% | 73 | 51% | 37 |  | 13 |  | 11 |  | 8 | 5 |
| Dak Lak | 6% | 74 | 30% | 22 | 49% | 11 |  | 4 |  | 3 |  | 2 | 2 |
| Ho Chi Minh | 18% | 212 | 63% | 134 | 51% | 69 |  | 24 |  | 21 |  | 15 | 9 |
| Dong Thap | 19% | 231 | 25% | 58 | 50% | 29 |  | 10 |  | 9 |  | 6 | 4 |
| **Total sample** |  | **1200** |  | **407** |  | **207** |  | **72** |  | **63** |  | **44** | **28** |
|  | **Rural - Male** | | | | | | | | | | | | |
|  | %Population of region | Sample size | %rural in region | Sample in rural cluster | %male | Sample male | %age 18-29 | Sample male age 18-29 | %age 30-44 | Sample male age 30-44 | %age 45-59 | Sample male age 45-59 | Sample male age 60+ |
| Hanoi | 23% | 272 | 66% | 180 | 49% | 88 | 36% | 32 | 35% | 31 | 21% | 18 | 7 |
| Hoa Binh | 13% | 154 | 82% | 126 | 50% | 63 |  | 23 |  | 22 |  | 13 | 5 |
| Phu Yen | 21% | 257 | 72% | 184 | 49% | 91 |  | 33 |  | 32 |  | 19 | 7 |
| Dak Lak | 6% | 74 | 70% | 52 | 50% | 26 |  | 9 |  | 9 |  | 5 | 3 |
| Ho Chi Minh | 18% | 212 | 37% | 78 | 49% | 38 |  | 14 |  | 13 |  | 8 | 3 |
| Dong Thap | 19% | 231 | 75% | 173 | 50% | 86 |  | 31 |  | 30 |  | 18 | 7 |
| **Total sample** |  | **1200** |  | **793** |  | **392** |  | **142** |  | **137** |  | **81** | **32** |
|  | **Rural - Female** | | | | | | | | | | | | |
|  | %Population of region | Sample size | %rural in region | Sample in rural cluster | %female | Sample female | %age 18-29 | Sample female age 18-29 | %age 30-44 | Sample female age 30-44 | %age 45-59 | Sample female age 45-59 | Sample female age 60+ |
| Hanoi | 23% | 272 | 66% | 180 | 51% | 92 | 31% | 29 | 31% | 29 | 22% | 20 | 14 |
| Hoa Binh | 13% | 154 | 82% | 126 | 50% | 63 |  | 20 |  | 20 |  | 14 | 9 |
| Phu Yen | 21% | 257 | 72% | 184 | 51% | 93 |  | 29 |  | 29 |  | 20 | 15 |
| Dak Lak | 6% | 74 | 70% | 52 | 50% | 26 |  | 8 |  | 8 |  | 6 | 4 |
| Ho Chi Minh | 18% | 212 | 37% | 78 | 51% | 40 |  | 12 |  | 12 |  | 9 | 7 |
| Dong Thap | 19% | 231 | 75% | 173 | 50% | 87 |  | 27 |  | 27 |  | 19 | 14 |
| **Total sample** |  | **1200** |  | **793** |  | **401** |  | **125** |  | **125** |  | **88** | **63** |

## Table II: Choice probabilities of the 10 DCE pairs of mildest health states

| State ID | Health state A | Health state B | Choice P |
| --- | --- | --- | --- |
| 187 | 12121 | 21111 | 87.80% |
| 188 | 12111 | 21121 | 78.57% |
| 189 | 11211 | 22111 | 80.43% |
| 190 | 11121 | 21211 | 64.10% |
| 191 | 11112 | 12221 | 93.02% |
| 192 | 11122 | 23111 | 52.27% |
| 193 | 11212 | 22112 | 82.93% |
| 194 | 12112 | 22211 | 77.27% |
| 195 | 12211 | 21112 | 58.14% |
| 196 | 11221 | 22122 | 88.37% |

## Table III. Descriptive analysis of C-TTO data

| **Profile** | **mean** | **SD** | **Median** | **IQR** | **Profile** | **mean** | **SD** | **Median** | **IQR** |
| --- | --- | --- | --- | --- | --- | --- | --- | --- | --- |
| **11112** | 0.9337 | 0.0933 | 0.95 | 0.1 | **31524** | 0.4836 | 0.3473 | 0.5 | 0.3 |
| **11121** | 0.9096 | 0.1145 | 0.95 | 0.1 | **31525** | 0.3907 | 0.4246 | 0.5 | 0.3 |
| **11122** | 0.8330 | 0.1878 | 0.9 | 0.15 | **32314** | 0.6452 | 0.2601 | 0.7 | 0.25 |
| **11211** | 0.9047 | 0.1134 | 0.95 | 0.1 | **32443** | 0.3382 | 0.4859 | 0.45 | 0.35 |
| **11212** | 0.8523 | 0.1218 | 0.9 | 0.15 | **33253** | 0.3702 | 0.4286 | 0.45 | 0.3 |
| **11221** | 0.8579 | 0.1075 | 0.9 | 0.15 | **34155** | 0.1651 | 0.4993 | 0.35 | 0.6 |
| **11235** | 0.5358 | 0.3542 | 0.6 | 0.35 | **34232** | 0.4948 | 0.3767 | 0.6 | 0.3 |
| **11414** | 0.5635 | 0.3614 | 0.65 | 0.3 | **34244** | 0.2934 | 0.4333 | 0.4 | 0.35 |
| **11421** | 0.6983 | 0.1781 | 0.7 | 0.2 | **34515** | 0.2835 | 0.4710 | 0.4 | 0.35 |
| **11425** | 0.5291 | 0.3179 | 0.6 | 0.25 | **35143** | 0.3209 | 0.4920 | 0.5 | 0.3 |
| **12111** | 0.9066 | 0.1354 | 0.95 | 0.1 | **35245** | 0.1953 | 0.4936 | 0.35 | 0.4 |
| **12112** | 0.8656 | 0.1331 | 0.9 | 0.15 | **35311** | 0.5635 | 0.3832 | 0.6 | 0.3 |
| **12121** | 0.8118 | 0.1845 | 0.85 | 0.25 | **35332** | 0.4427 | 0.3835 | 0.55 | 0.25 |
| **12244** | 0.4610 | 0.4035 | 0.55 | 0.3 | **42115** | 0.4538 | 0.4169 | 0.6 | 0.3 |
| **12334** | 0.5377 | 0.3461 | 0.65 | 0.25 | **42321** | 0.5657 | 0.3371 | 0.6 | 0.25 |
| **12344** | 0.4466 | 0.3922 | 0.55 | 0.4 | **43315** | 0.4160 | 0.3987 | 0.5 | 0.25 |
| **12513** | 0.6072 | 0.2292 | 0.65 | 0.25 | **43514** | 0.3816 | 0.3542 | 0.45 | 0.25 |
| **12514** | 0.4791 | 0.4371 | 0.6 | 0.25 | **43542** | 0.1592 | 0.4888 | 0.35 | 0.7 |
| **12543** | 0.2466 | 0.5231 | 0.4 | 0.35 | **43555** | -0.0361 | 0.4948 | 0.1 | 0.85 |
| **13122** | 0.7308 | 0.2340 | 0.8 | 0.15 | **44125** | 0.3525 | 0.3412 | 0.4 | 0.25 |
| **13224** | 0.6013 | 0.3734 | 0.7 | 0.25 | **44345** | 0.0284 | 0.5053 | 0.2 | 0.9 |
| **13313** | 0.7263 | 0.2069 | 0.75 | 0.2 | **44553** | -0.0992 | 0.4675 | 0.05 | 0.8 |
| **14113** | 0.7180 | 0.2464 | 0.75 | 0.1 | **45133** | 0.3105 | 0.4790 | 0.5 | 0.3 |
| **14554** | 0.0441 | 0.4863 | 0.2 | 0.65 | **45144** | 0.1673 | 0.5056 | 0.3 | 0.4 |
| **15151** | 0.3742 | 0.4730 | 0.5 | 0.35 | **45233** | 0.3004 | 0.4559 | 0.4 | 0.4 |
| **21111** | 0.9252 | 0.1266 | 0.95 | 0.1 | **45413** | 0.3239 | 0.3918 | 0.4 | 0.25 |
| **21112** | 0.8619 | 0.1451 | 0.9 | 0.15 | **51152** | 0.1701 | 0.5049 | 0.35 | 0.7 |
| **21315** | 0.6377 | 0.2926 | 0.7 | 0.25 | **51451** | 0.1598 | 0.5146 | 0.3 | 0.45 |
| **21334** | 0.5662 | 0.2929 | 0.6 | 0.25 | **52215** | 0.3189 | 0.4835 | 0.45 | 0.3 |
| **21345** | 0.4313 | 0.3668 | 0.5 | 0.35 | **52335** | 0.2235 | 0.4759 | 0.4 | 0.3 |
| **21444** | 0.2971 | 0.4394 | 0.4 | 0.4 | **52431** | 0.3094 | 0.4218 | 0.4 | 0.3 |
| **22434** | 0.3893 | 0.4365 | 0.5 | 0.35 | **52455** | -0.1038 | 0.4908 | -0.025 | 0.85 |
| **23152** | 0.4977 | 0.3592 | 0.55 | 0.3 | **53221** | 0.5449 | 0.2552 | 0.6 | 0.25 |
| **23242** | 0.4570 | 0.4320 | 0.55 | 0.3 | **53243** | 0.1377 | 0.5140 | 0.325 | 0.8 |
| **23514** | 0.4105 | 0.4526 | 0.5 | 0.25 | **53244** | 0.0758 | 0.5232 | 0.25 | 0.9 |
| **24342** | 0.3522 | 0.4204 | 0.5 | 0.3 | **53412** | 0.3461 | 0.4217 | 0.45 | 0.25 |
| **24443** | 0.2377 | 0.4728 | 0.4 | 0.35 | **54153** | 0.0213 | 0.5152 | 0.2 | 0.85 |
| **24445** | 0.0817 | 0.4925 | 0.3 | 0.7 | **54231** | 0.3323 | 0.4706 | 0.5 | 0.3 |
| **24553** | 0.0410 | 0.5208 | 0.25 | 0.95 | **54342** | 0.1775 | 0.4339 | 0.3 | 0.45 |
| **25122** | 0.5864 | 0.3607 | 0.65 | 0.3 | **55225** | 0.0785 | 0.4734 | 0.225 | 0.7 |
| **25222** | 0.5684 | 0.3080 | 0.65 | 0.25 | **55233** | 0.1436 | 0.5088 | 0.3 | 0.75 |
| **25331** | 0.4988 | 0.3341 | 0.55 | 0.3 | **55424** | 0.1266 | 0.4490 | 0.25 | 0.425 |
| **31514** | 0.4885 | 0.3780 | 0.55 | 0.3 | **55555** | -0.5028 | 0.4211 | -0.6 | 0.5 |
